# Solid pseudopapillary neoplasms of the pancreas are dependent on the Wnt pathway

**DOI:** 10.1002/1878-0261.12490

**Published:** 2019-07-03

**Authors:** Pier Selenica, Nitya Raj, Rahul Kumar, David N. Brown, Oriol Arqués, Diane Reidy, David Klimstra, Matija Snuderl, Jonathan Serrano, Héctor G. Palmer, Britta Weigelt, Jorge S. Reis‐Filho, Maurizio Scaltriti

**Affiliations:** ^1^ Department of Pathology Memorial Sloan Kettering Cancer Center New York NY USA; ^2^ Department of Medicine Memorial Sloan Kettering Cancer Center New York NY USA; ^3^ Department of Pathology New York University Langone Medical Center and Medical School NY USA; ^4^ Stem Cells and Cancer Laboratory Vall d'Hebron Institute of Oncology (VHIO) Barcelona Spain; ^5^ CIBERONC Madrid Spain; ^6^ Human Oncology & Pathogenesis Program (HOPP) Memorial Sloan Kettering Cancer Center New York NY USA

**Keywords:** beta‐catenin, gene expression, methylation, pancreas, SPN, Wnt

## Abstract

Solid pseudopapillary neoplasms (SPNs) are rare and relatively indolent tumors of the pancreas. While primary SPNs can be surgically resected, there are currently no therapies available for patients with advanced stage disease. Given that these tumors frequently carry *CTNNB1* hotspot (recurrently mutated loci in a gene) mutations resulting in β‐catenin nuclear accumulation, it has been speculated that the Wnt pathway may be a driver in this disease. Here, we present a comprehensive “multi‐omics” study where the genome, transcriptome, and methylome of SPNs were analyzed. We found that SPNs are characterized by a low‐complexity genome where somatic mutations in *CTNNB1*, present in 100% of the cases, are the only actionable genomic lesions. Compared to more common subtypes of pancreatic tumors (adenocarcinomas and pancreatic neuroendocrine tumors), SPNs show high expression levels of genes belonging to the Wnt pathway. Their methylome was consistent with an epithelial cell origin and a general upregulation of Wnt pathway genes. Clinical studies to evaluate the exquisite sensitivity of SPNs to inhibitors of the Wnt pathway are warranted.

**Abbreviations**
ADC: adenocarcinoma
ENCODE: encyclopedia of DNA elements
indel: small insertion and deletion
PNET: pancreatic neuroendocrine tumors
SNV: single nucleotide variant
SPN: solid pseudopapillary neoplasms

## 1 Introduction

Solid pseudopapillary neoplasms (SPNs) are rare pancreatic tumors of unknown etiology, accounting for ~ 1% of all pancreatic exocrine neoplasms. SPNs typically present as large, solitary, well‐circumscribed lesions with indolent clinical course and a low propensity to metastasize (Klimstra *et al*., 2000; Ren *et al*., 2014). Although they are commonly managed surgically, some SPNs can exhibit a more aggressive behavior and metastasize. Currently, there is no consensus on effective systemic treatments for malignant SPNs, and given the rarity of advanced disease, prospective clinical trials testing novel therapies have not been performed as yet.

Solid pseudopapillary neoplasms are characterized by the presence of somatic *CTNNB1* exon 3 hotspot mutations (Abraham *et al*., 2002; Guo *et al*., 2017; Tanaka *et al*., 2001; Wu *et al*., 2011), leading to stabilization and nuclear localization of β‐catenin, which can be detected by immunohistochemistry (Tanaka *et al*., 2001). Given these premises, the role of the Wnt pathway in SPN development has been suggested. The Wnt pathway is a signal transduction cascade that under normal physiological conditions regulates development and stemness but has also been tightly associated with multiple growth‐related pathologies and cancer (Nusse and Clevers, 2017). A key component of the Wnt pathway is the protein β‐catenin encoded by the gene *CTNNB1*, which regulates transcription of downstream target genes (e.g., *LEF1*,* AXIN2*). Hotspot mutations in *CTNNB1* can cause abnormal accumulation of nuclear β‐catenin through stabilization of the protein and eventually constitutive activation of the pathway.

Here, we assessed whether SPNs are dependent on the Wnt signaling pathway and performed a comprehensive “multi‐omics” profiling of SPNs collected at our institution during the last two decades to define their repertoire of somatic mutations and copy number alterations, the transcriptional activation of the Wnt pathway and their methylation profiles.

## 2 Methods

### 2.1 Cases

Patients with written informed consent for research that had a diagnosis of SPN treated at Memorial Sloan Kettering Cancer Center (MSKCC) were identified from our institutional database. Clinicopathological characteristics of the SPN patients are shown in Table 1. Electronic medical records were retrospectively reviewed for data on patient demographics, pathology, presentation (benign versus malignant SPN), treatment (surgical and systemic therapies), and outcomes. In addition, pancreatic adenocarcinomas (ADCs) and pancreatic neuroendocrine tumors (PNETs) were identified as controls. All pathology was reviewed by gastrointestinal pathologists at MSKCC experienced in the diagnosis of SPN. Approval for data collection and analysis was obtained from the MSKCC Institutional Review Board. The study conforms to the guidelines set by the Declaration of Helsinki.

**Table 1:** Baseline patient characteristics

SPNs	All *n* = 14 *N* (%)
Age, mean (range), years	37 (22–71)
Sex	
Female	11 (79)
Male	3 (21)
Smoker/prior smoker	1 (7)
Symptomatic at presentation	14 (100)
Pain	11 (79)
Diarrhea	2 (14)
Other	1 (7)
Primary tumor location	
Head	3 (21)
Body	5 (36)
Tail	6 (43)
Primary tumor size, mean (range), centimeters	5.9 (1.2–11)
Primary tumor resected	14 (100)
Surgery	
Pancreaticoduodenectomy	3 (21)
Distal pancreatectomy	11 (79)
Hepatic debulking	1 (7)
Metastatic disease	3 (21)
Location of metastases	
Liver	2 (14)
Omentum/peritoneum	2 (14)
Lung	1 (7)
Lymph nodes	1 (7)
Systemic therapy	1 (7)
Hepatic arterial embolization	1 (7)

### 2.2 Immunohistochemistry

Immunohistochemical analysis for β‐catenin (Clone 14, 1 : 200; BD Transduction, San Jose, CA) was performed on 4‐μm‐thick sections from representative formalin‐fixed, paraffin‐embedded SPN tissue blocks, as previously described (Basturk *et al*., 2016). Positive and negative controls were included in each slide run. The expression of β‐catenin was assessed in the membrane and cytoplasmic and nuclear subcellular compartments, and considered abnormal if cytoplasmic and nuclear accumulation were present.

### 2.3 Whole‐exome sequencing analysis

DNA samples extracted from 14 flash‐frozen SPNs and matched normal tissues were subjected to whole‐exome sequencing at the MSKCC Integrated Genomics Operation as previously described (Ng *et al*., 2017). Briefly, raw sequence reads were aligned to the reference human genome GRCh37 using the burrows‐wheeler aligner (BWA 0.7.15) (Li and Durbin, 2009). Local realignment, duplicate read removal, and base quality score recalibration were performed using the genome analysis toolkit (GATK 3.7) (McKenna *et al*., 2010). Somatic single nucleotide variants (SNVs) were called using mutect (1.1.7) (Cibulskis *et al*., 2013), and small insertions and deletions (indels) were identified using strelka (1.0.15) (Saunders *et al*., 2012), varscan2 (2.3.7) (Koboldt *et al*., 2012), lancet (1.0.0) (Narzisi *et al*., 2018), and scalpel (0.5.3) (Narzisi *et al*., 2014) and further curated by manual inspection. SNVs and indels outside of target regions were filtered out, as were SNVs and indels for which the variant allele fraction (VAF) in the tumor sample was < 5 times that of the paired normal VAF as previously described (Ng *et al*., 2017; Weigelt *et al*., 2018). Finally, SNVs and indels found at > 5% global minor allele frequency in dbSNP (build 137) and > 5% global allele frequency in exac (0.3.1) were discarded. Somatic copy number alterations and loss of heterozygosity were obtained using facets (Shen and Seshan, 2016). The cancer cell fractions (CCF) of all mutations were computed using absolute (1.0.6) (Carter *et al*., 2012). A mutation was classified as clonal if its probability of being clonal was > 50% (Landau *et al*., 2013) or if the lower bound of the 95% confidence interval of its CCF was > 90% (Ng *et al*., 2017; Weigelt *et al*., 2018). Mutations that did not meet the above criteria were considered subclonal. A combination of *in silico* functional predictors was used to define the potential functional impact of each missense SNV as previously described (Martelotto *et al*., 2014; Ng *et al*., 2017). Mutation hotspots were assigned according to Chang *et al*. (2016).

*CTNNB1* mutation frequencies were assessed in 10 100 common cancers from The Cancer Genome Atlas (TCGA) (Bailey *et al*., 2018; Gao *et al*., 2013), including 175 pancreatic ADCs. In addition, mutational data from 8 SPNs from Wu *et al*. (2011) and 344 non‐SPN pancreatic tumors, including 23 acinar cell carcinomas from Jiao *et al*. (2014), 98 PNETs from Scarpa *et al*. (2017), and 24 cystic tumors of the pancreas Wu *et al*. (2011). The mutational data were retrieved from cBioPortal (Gao *et al*., 2013). *CTNNB1* mutation and hotspot mutation frequencies were assessed following exclusion of hypermutated cases, defined as cancers harboring more than 1000 nonsynonymous mutations, microsatellite‐unstable, or harboring *POLE* or *POLD1* exonuclease domain mutations, as previously described (Pareja *et al*., 2018). Mutation diagrams (‘lollipop’ plots) were generated using MutationMapper on cbioportal (Gao *et al*., 2013) and manually curated.

### 2.4 Gene expression analysis

Total RNA extracted from nine SPNs, seven pancreatic ADCs, and 8 PNETs was subjected to expression analysis of 240 selected genes using the nCounter platform from Nanostring Technologies, Seattle, WA (Table S3). The raw counts were normalized using the nSolver analysis software. We performed background subtraction using the negative control and normalized the background corrected counts using the housekeeping genes *ACTB*,* MRPL19*,* PSMC4*,* RPLP0*, and *SF3A1*. Comparison between SPN samples and ADCs or PNETs was done using the r/bioconductor limma package, and genes with *P* values < 0.05 were considered as significantly differentially expressed.

### 2.5 Genome‐wide methylation analysis

DNA extracted from 13 SPNs was subjected to Illumina Infinium Methylation 850K array profiling as previously described (Chiang *et al*., 2016). The r/bioconductor minfi package was used to process the raw files and obtain the normalized beta values. The methylation data in the form of beta values of cells of epithelial (*n* = 22), fibroblast (*n* = 10), and muscle cell (*n* = 6) origin were downloaded from encyclopedia of DNA elements (ENCODE) project (ENCODE Project Consortium *et al*., 2007) and processed as described above. To avoid the confounding effects of gender, probes mapping to sex chromosomes were discarded while batch effect was removed using the combat method (Johnson *et al*., 2007) implemented in the r/bioconductor package sva. Probes which were differentially methylated between ENCODE samples of epithelial, fibroblast, and muscle cell origin were identified using the Kruskal–Wallis test and an FDR‐adjusted *P*‐value < 0.1 (Table S4). The SPN methylation data were projected onto the first two principal components estimated using the ENCODE samples. Probes of the Wnt pathway found to be differentially methylated between SPNs and ENCODE samples were subjected to hierarchical cluster analysis using Pearson correlation and Ward's distance.

### 2.6 Statistical analysis

Using the transcriptomic data, genes differentially expressed between SPNs and ADCs or PNETs were identified using a linear model fitted to the expression data. We considered genes with a *P*‐value < 0.05 and an absolute fold change value > 2 as statistically significant. In the case of methylation data, the data were filtered by identifying probes with multimodal distributions of beta values in the ENCODE dataset. A mixture of Gaussian distributions was fitted to the pooled data using the r/bioconductor package mclust. Probes which were differentially methylated between ENCODE samples of epithelial, fibroblast, and muscle cell origin were identified using the Kruskal–Wallis test and an FDR‐adjusted *P*‐value < 0.1. The SPN methylation data were projected onto the first two principal components estimated using the ENCODE samples. All hypothesis tests were unpaired, and *P*‐values were two‐sided. Unless otherwise stated, all computations were performed in r/bioconductor.

## 3 Results

### 3.1 Landscape of somatic mutations in SPNs

**Figure 1:**
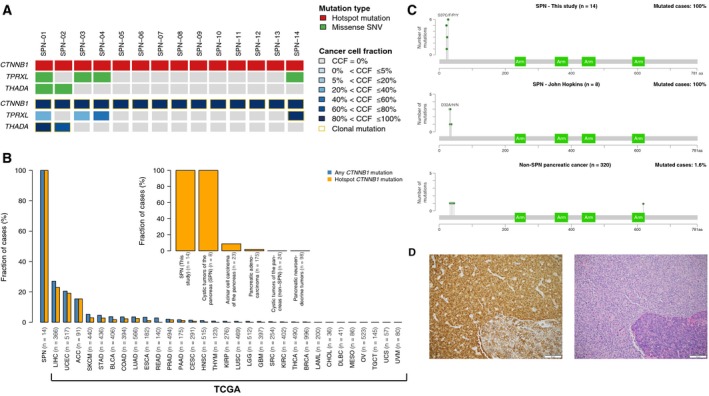
Prevalence of *CTNNB1* mutations in SPNs and other common cancer types. (A) Mutation type (top) and CCF (bottom) of all recurrently mutated genes in SPNs subjected to whole‐exome sequencing in this study. (B) Bar plots depicting the frequency of *CTNNB1* and *CTNNB1* hotspot mutations in 14 SPNs from this study and in 10 100 samples from 31 common cancer types from TCGA. The insert shows the prevalence of *CTNNB1* hotspot mutations in SPNs analyzed in this study compared to SPNs and other pancreatic tumors from this study and from TCGA. (C) Lollipop plot representing the β‐catenin protein domains and the spectra of the *CTNNB1* mutations in 14 SPNs profiled in this study (top), in eight SPNs from Wu *et al*. (2011) (middle), and in 320 non‐SPN pancreatic tumors available from cBioPortal (Gao *et al*., 2013). Mutations are shown on the *x*‐axis, and the frequency of a particular mutation is represented by the height of each “lollipop” (*y*‐axis). (D) Representative micrographs of an SPN with adjacent normal tissue (bottom right in both images). Hematoxylin‐ and eosin‐stained section (left) and β‐catenin immunohistochemical analysis (right).

Whole‐exome sequencing analysis revealed clonal somatic hotspot *CTNNB1* mutations in all 14 SPNs analyzed (Fig. 1A). Only two additional recurrently mutated genes were identified in more than one patient sample (*THADA* and *TPRXL*; Fig. 1A, Tables [Link], [Link]). All SPNs displayed low levels of copy number alterations, consistent with a lack of genomic instability characteristic of SPNs (Fig. S1). A Pan‐Cancer analysis of 10 100 nonhypermutated cancers across 31 common cancer types from TCGA (Bailey *et al*., 2018; Gao *et al*., 2013) revealed that while *CTNNB1* exon 3 mutations were present at 15–20% of liver, uterine, and adrenocortical carcinomas, mutations affecting *CTNNB1* exon 3 are rare in other cancer types (< 3%; Fig. 1B). Furthermore, of the 328 pancreatic tumors included in the cBioPortal database (Bailey *et al*., 2018; Gao *et al*., 2013; Jiao *et al*., 2014; Scarpa *et al*., 2017; Wu *et al*., 2011) only 12 (3.7%) harbored *CTNNB1* exon 3 mutations (Fig. 1C). Eight of these 12 cases were SPNs, whereas the remaining tumors bearing *CTNNB1* hotspot mutations were ADCs (*n* = 2) or pancreatoblastomas (*n* = 2) (Wu *et al*., 2011). Thus, we conclude that in the context of pancreatic neoplasms in adults, mutations in β‐catenin (amino acids 32 through 45) strongly prompt a diagnosis of SPN. SPNs displayed low mutational burdens (median: 19, range: 7–50) compared to β‐catenin wild‐type pancreatic tumors (median: 50, range: 1–185; *P* < 0.0001, Mann–Whitney *U*) (Bailey *et al*., 2018; Gao *et al*., 2013; Jiao *et al*., 2014; Scarpa *et al*., 2017; Wu *et al*., 2011), which is consistent with the finding that a subset of endometrioid endometrial carcinomas driven by *CTNNB1* mutations and Wnt pathway activation are characterized by a relatively low tumor mutational burden (Liu *et al*., 2014).

### 3.2 Activation of the Wnt pathway in SPNs

**Figure 2:**
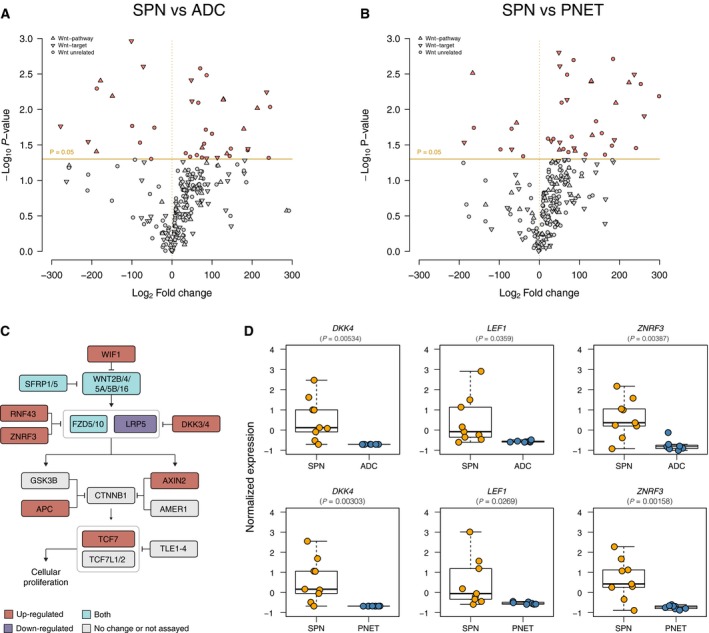
Patterns of gene expression in SPNs, pancreatic ADCs, PNETs, and normal tissue samples. (A) Volcano plot showing the genes differentially expressed between SPNs and ADCs. The Log_2_ fold change refers to the ratio of mean expression between SPNs and ADCs. Orange line depicts *P*‐value cut‐off of 0.05. (B) Volcano plot showing the genes differentially expressed genes between SPNs and PNETs. The Log_2_ fold change refers to the ratio of mean expression between SPNs and PNETs. Orange line depicts *P*‐value cut‐off of 0.05. (C) Schematic illustration of the main components of the canonical Wnt pathway. The genes found to be significantly differentially expressed (*P* < 0.05) in (A) and (B) are color‐coded according to the legend. (D) Boxplots depicting the expression levels of the Wnt pathway genes *DKK4*,*LEF1,* and *ZNRF3* in SPNs, ADCs, and PNETs. Modified *t*‐test.

From a genomics standpoint, SPNs appear to be driven solely by *CTNNB1* activating somatic mutations, which result in nuclear accumulation of the protein (Polakis, 1999). Accordingly, we detected strong β‐catenin protein expression in both cytosol and nuclei of all SPNs analyzed (Fig. 1D). This, in turn, initiates a transcriptional program primed to activate the Wnt pathway (Mosimann *et al*., 2009). To investigate the components of the Wnt signaling pathway activated in SPNs, we performed a differential gene expression analysis of nine SPNs, four of which were metastatic, seven pancreatic ADCs, and eight PNETs using the Nanostring platform (Table S3). Compared to ADCs and PNETs, SPNs displayed significantly higher expression levels of genes encoding components of the Wnt pathway, including *DKK4*, which has recently been detected by mass spectrometry in SPNs (Park *et al*., 2015), and direct TCF/β‐catenin target genes such as *LEF1* (Aoki *et al*., 1999) or *AXIN2* (Jho *et al*., 2002) (Fig. 2A–D, Fig. S2). Other genes whose expression has been described to be induced by Wnt/β‐catenin signaling activation such as *LGR5*,* ASCL2,* or *CCND1* were also expressed at significantly higher levels in SPNs (Fig. S2).

### 3.3 Methylome analysis of SPNs

**Figure 3:**
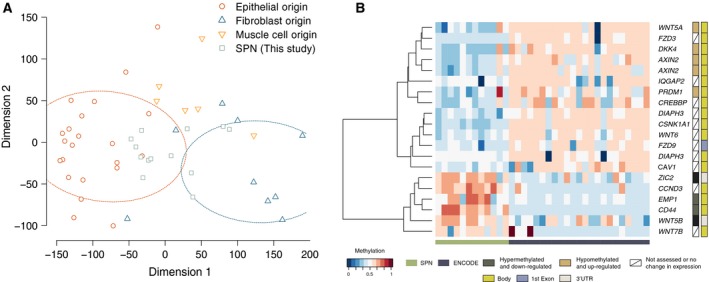
Patterns of methylation in SPNs and normal tissue samples. (A) Principal component analysis of methylation data showing the clustering of samples of different histological origins from the ENCODE project together with SPNs profiled in this study. (B) Heatmap of methylation data from SPN samples profiled in this study and ENCODE samples of epithelial origin. Probes found to be differentially methylated (FDR < 0.1) between SPN and ENCODE samples were mapped to the Wnt pathway. With few exceptions, the probes mapped to gene bodies where hypomethylation corresponded to upregulation of the corresponding transcript. The two exceptions were probes mapping to *ZIC2* and *WNT5B* which were located in 3′UTR regions where hypermethylation also corresponded to upregulation of the upstream gene.

Given the unknown histogenesis of SPNs, we analyzed the global methylation profiles of 12 of the 14 SPNs analyzed by whole‐exome sequencing and compared their methylation patterns with those of normal cells of various anatomical sites from the ENCODE database (ENCODE Project Consortium *et al*., 2007). The majority of the SPNs profiled in this study clustered with epithelial cells (Fig. 3A). We further performed a differential analysis between ENCODE samples of epithelial origin and SPNs, which identified 2511 differentially methylated probes (Table S3). Probes that were significantly differentially methylated between samples of epithelial, fibroblast, and muscle cell origin in the ENCODE database were mapped to genes in the *Wnt* pathway and showed differential methylation in the gene bodies (intronic and exonic areas of a gene), which, based on ontology, would have resulted in upregulation of the respective transcript (e.g. *WNT5A*). Conversely, hypermethylated probe sets corresponded with genes whose transcripts were downregulated in SPNs (e.g., *CD44*; Fig. 3B). Two exceptions were probes mapping to *ZIC2* and *WNT5B,* which were found to be hypermethylated in SPN compared to ENCODE samples. Unlike *CD44*, these genes had a significantly higher expression in SPNs compared to ADCs or PNETs. Also, the methylation pattern is not unexpected, given that the hypermethylated probes mapped to the 3′UTR of the corresponding gene, a phenomenon not uncommonly associated with overexpression of the target gene.

## 4 Discussion

Here, we report a multi‐omics analysis of SPNs compared to the other two more common subtypes of pancreatic cancer, ADC and PNET. In agreement with the existing literature, we found that SPNs have low genomic complexity and are characterized by the invariable presence of *CTNNB1* exon 3 mutations. Accordingly, we found consistent increased expression of genes encoding components of the Wnt pathway in these tumors. Nuclear β‐catenin accumulation promotes the expression of a panel of Wnt target genes codifying for proteins involved in a myriad of cell processes. Among them, some codify components of the Wnt/β‐catenin pathway itself such as *LEF1*,* AXIN2*, or *RNF43*, which induce a positive feedback loop that enhances signaling activation (Zhan *et al*., 2017). Here, we observed that SPNs with mutations in *CTNBB1* that provoke constitutive accumulation of nuclear β‐catenin express higher levels of some of these Wnt target genes that are also Wnt pathway components such as *LEF1*,* AXIN2*, and *RNF43* (Fig. 3).

We also observed that other genes codifying components of the Wnt pathway are highly expressed in SPN than ADC, such as ligands (*WNT5A*,* WNT2B*, or *RSPO4*), receptor components (*LRG6* or *FZD10*), or other key proteins for signal transduction such as APC. Although these are not formally demonstrated to be Wnt/β‐catenin pathway target genes, their higher expression could suggest and enhanced activation of the pathway additional to that promoted by *CTNNB1*/β‐catenin activating mutations.

The lack of somatic genetic alterations other than *CTNNB1* mutations and low levels of genomic complexity may provide an explanation for the indolent behavior of SPNs, akin to colorectal polyps associated with familial adenomatous polyposis in patients with germline *APC* mutations developing multiple *KRAS* wild‐type, benign tumors where a dysfunctional APC can be the only driver (Leoz *et al*., 2015; Takane *et al*., 2016). In mice, activation of β‐catenin is indeed sufficient to produce large pancreatic tumors that resemble human SPNs both morphologically and by immunohistochemical analysis (Heiser *et al*., 2008).

Of note, a recent report identified also inactivating mutations of the epigenetic regulators *KDM6A*,* TET1*, and *BAP1* to be associated with metastatic spread (Amato *et al*., 2019). Whether these alterations are also playing a role in regulating the expression of Wnt‐dependent genes remains to be elucidated.

While inhibition of the Wnt pathway may be an intuitive therapeutic option for this disease, its rarity and the lack of available preclinical models are clear limitations in the advancement toward an effective treatment for metastatic SPNs. Furthermore, the more clinically advanced Wnt pathway inhibitors target components that are upstream of β‐catenin transcriptional activity and would therefore be futile for the treatment of SPN patients.

## 5 Conclusions

Taken together, our data suggest that SPNs have simple genomes and appear to be uniformly driven by *CTNNB1* exon 3 mutations with consequent activation of the Wnt signaling. Future drugs designed to target directly β‐catenin may have therapeutic potential in this disease.

## Conflict of interest

MS received honoraria from ADC Pharma and Menarini Ricerche; research funds received from Puma Biotechnology, Daiichi‐Sankyo, Immunomedics, and Menarini Ricerche; is a co‐founder of Medendi Medical Travel; and is in the scientific board of the Bioscience Institute. JSR‐F reports personal/ consultancy fees from VolitionRx, Page.AI, Invicro, Roche, Genentech, Ventana and Goldman Sachs, outside the submitted work.

## Author contributions

JSR‐F and MS conceived the study. DK performed the pathology review. RK, PS, and DNB performed bioinformatics analyses. NR provided the tumor samples with their clinical annotation; HP and OA performed and interpreted the nanostring analysis. MSn and JS performed the methylation analysis. PS, DNB, BW, NR, JSR‐F, and MS interpreted the data. PS, DNB, BW, JSR‐F, and MS wrote the first draft of the manuscript, which was edited and approved by all authors.

## Supporting information


**Fig. S1**. Copy number profiles of SPNs analyzed in this study.
**Fig. S2**. Differential gene expression analysis of SPNs, ADCs and PNETs.
**Table S1**. Whole‐exome sequencing statistics.

**Table S2**. Nonsynonymous somatic mutations identified in SPNs using whole‐exome sequencing.

**Table S3**. Genes in the Wnt pathway assessed by Nanostring nCounter.

**Table S4**. Probes differentially methylation between SPN samples (this study) and ENCODE samples of epithelial origin.
