# Supplementary material for: Solid pseudopapillary neoplasms of the pancreas are dependent on the Wnt pathway
Source: Mol Oncol. 2019 Jul 3;13(8):1684–92. doi: 10.1002/1878-0261.12490 (PMC6670010; doi:10.1002/1878-0261.12490)
Supplement: Supplementary file 1 — Fig. S1. Copy number profiles of SPNs analyzed in this study. Fig. S2. Differential gene expression analysis of SPNs, ADCs and PNETs. Table S1. Whole‐exome sequencing statistics. [file MOL2-13-1684-s001.docx]

**SUPPLEMENTARY MATERIALS**

**Solid pseudopapillary neoplasms of the pancreas are dependent on the Wnt pathway**

Selenica et al.

**SUPPLEMENTARY FIGURES**

**Supplementary Figure 1**


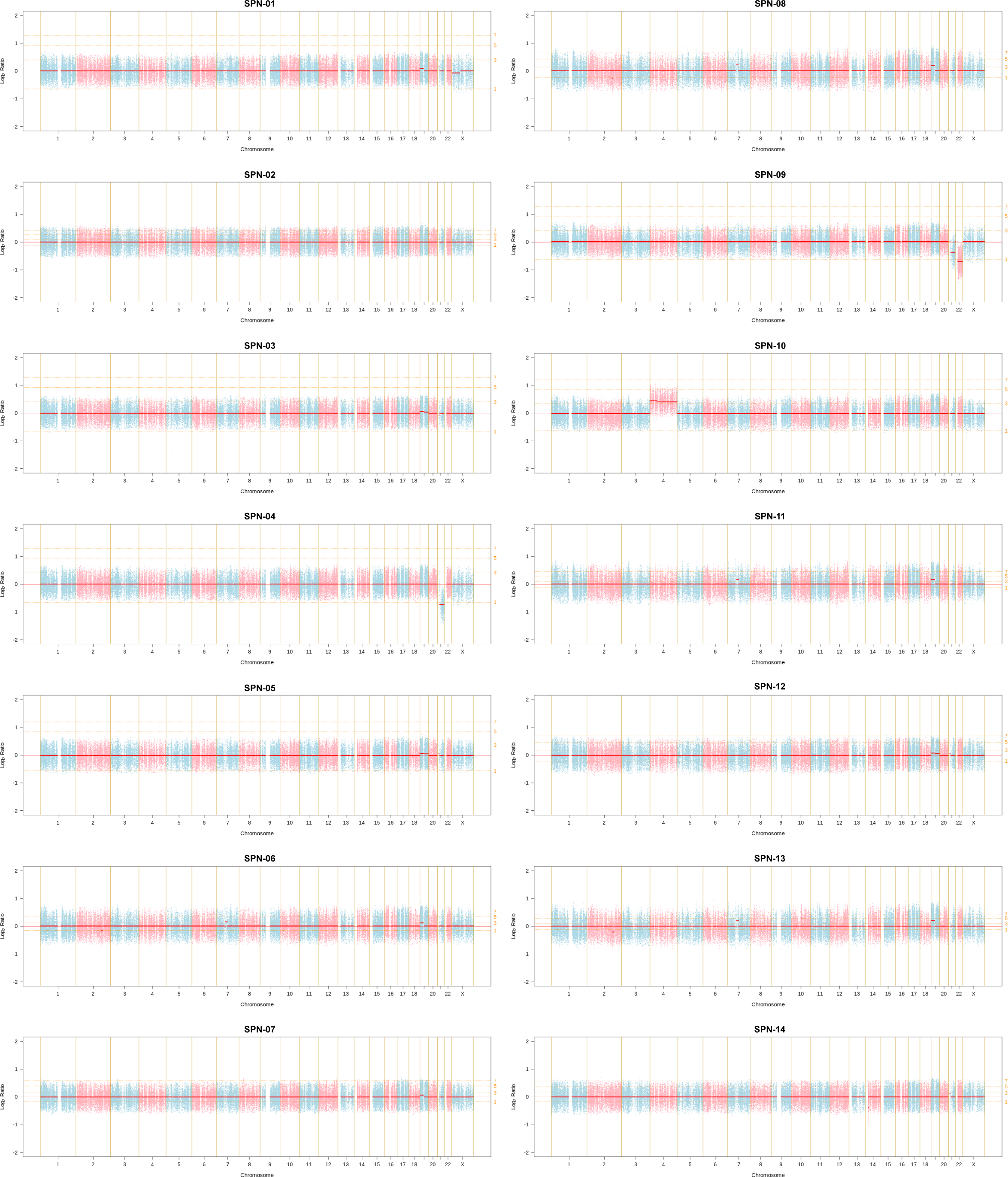


**Supplementary Figure 1. Copy number profiles of SPNs analyzed in this study.**

Genome-wide Log_2_ ratio profiles of SPNs subjected to whole-exome sequencing. In each panel, the segmented Log_2_ ratios are plotted on the y-axis and chromosomes along the x-axis, color-coded in red and blue. Copy numbers are depicted by horizontal orange lines, and the number of copies are shown on the right y-axis.

**Supplementary Figure 2**

**Supplementary Figure 2. Differential gene expression analysis of SPNs, ADCs and PNETs.**

(A) Boxplots showing the expression levels of *AXIN2*, *ASCL2* and *FZD10* in SPNs, ADCs and PNETs. Modified t-test. (B) Hierarchical clustering analysis of the significant differentially expressed genes between SPNs, ADCs and PNETs (P < 0.05), using Ward’s distance and 1-Pearson correlation.

**SUPPLEMENTARY TABLES**

Supplementary Table 1. Sequencing statistics

| **Sample ID** | **Target Territory** | **Total Reads** | **Percent Selected Bases** | **Mean Target Coverage** | **Percent Target Bases 10X** | **Percent Target Bases 20X** | **Percent Target Bases 30X** | **Percent Target Bases 40X** | **Percent Target Bases 50X** | **Percent Target Bases 100X** |
| --- | --- | --- | --- | --- | --- | --- | --- | --- | --- | --- |
| SPN-01-N | 51189318 | 61373845 | 92.44% | 99.29 | 98.76% | 96.83% | 93.63% | 89.01% | 82.96% | 43.99% |
| SPN-01-T | 51189318 | 140035644 | 84.98% | 197.45 | 99.51% | 99.03% | 98.32% | 97.32% | 96.02% | 84.11% |
| SPN-02-N | 51189318 | 82695535 | 89.90% | 126.44 | 99.19% | 98.11% | 96.41% | 94.02% | 90.75% | 62.37% |
| SPN-02-T | 51189318 | 160556663 | 76.62% | 203.13 | 99.51% | 99.04% | 98.39% | 97.51% | 96.37% | 85.80% |
| SPN-03-N | 51189318 | 58486985 | 74.84% | 74.47 | 98.47% | 95.40% | 89.99% | 81.97% | 71.86% | 23.17% |
| SPN-03-T | 51189318 | 166022466 | 93.26% | 264.22 | 99.51% | 99.14% | 98.71% | 98.16% | 97.47% | 91.60% |
| SPN-04-N | 51189318 | 66001621 | 90.45% | 101.62 | 99.05% | 97.31% | 94.40% | 90.14% | 84.41% | 45.80% |
| SPN-04-T | 51189318 | 134306513 | 90.19% | 205.94 | 99.58% | 99.13% | 98.43% | 97.46% | 96.23% | 85.24% |
| SPN-05-N | 51189318 | 47154971 | 88.47% | 72.42 | 98.35% | 95.04% | 89.22% | 80.62% | 70.02% | 21.47% |
| SPN-05-T | 51189318 | 141621398 | 79.35% | 186.91 | 99.51% | 98.95% | 98.15% | 97.06% | 95.67% | 82.70% |
| SPN-06-N | 51189318 | 76976800 | 90.40% | 119.21 | 99.01% | 97.70% | 95.58% | 92.52% | 88.39% | 56.88% |
| SPN-06-T | 51189318 | 130939094 | 79.13% | 172.43 | 99.54% | 98.91% | 97.92% | 96.57% | 94.83% | 78.71% |
| SPN-07-N | 51189318 | 130277313 | 85.74% | 185.49 | 99.55% | 99.03% | 98.25% | 97.15% | 95.72% | 82.24% |
| SPN-07-T | 51189318 | 111374009 | 84.07% | 163.65 | 99.47% | 98.82% | 97.83% | 96.43% | 94.58% | 77.22% |
| SPN-08-N | 51189318 | 66464140 | 79.31% | 92.34 | 98.96% | 96.83% | 93.18% | 87.81% | 80.79% | 38.41% |
| SPN-08-T | 51189318 | 153705274 | 77.71% | 202.14 | 99.55% | 99.06% | 98.37% | 97.42% | 96.21% | 85.09% |
| SPN-09-N | 51189318 | 67443968 | 90.32% | 106.40 | 99.08% | 97.36% | 94.50% | 90.38% | 84.99% | 48.71% |
| SPN-09-T | 51189318 | 143502013 | 89.55% | 219.19 | 99.54% | 99.09% | 98.44% | 97.56% | 96.42% | 86.47% |
| SPN-10-N | 51189318 | 54469465 | 91.84% | 88.54 | 98.81% | 96.58% | 92.72% | 86.97% | 79.47% | 35.45% |
| SPN-10-T | 51189318 | 128952625 | 90.73% | 205.98 | 99.58% | 99.13% | 98.49% | 97.58% | 96.40% | 85.51% |
| SPN-11-N | 51189318 | 67446407 | 89.82% | 104.24 | 99.14% | 97.57% | 94.94% | 91.01% | 85.66% | 47.71% |
| SPN-11-T | 51189318 | 137519948 | 67.50% | 151.32 | 99.51% | 98.86% | 97.77% | 96.20% | 94.10% | 73.53% |
| SPN-12-N | 51189318 | 62150080 | 80.40% | 92.45 | 98.63% | 96.57% | 93.16% | 88.14% | 81.45% | 39.28% |
| SPN-12-T | 51189318 | 141174586 | 67.95% | 170.44 | 99.10% | 98.43% | 97.47% | 96.17% | 94.50% | 78.85% |
| SPN-13-N | 51189318 | 61320774 | 88.98% | 94.65 | 98.97% | 97.02% | 93.71% | 88.71% | 82.03% | 40.23% |
| SPN-13-T | 51189318 | 146818768 | 91.20% | 226.72 | 99.63% | 99.27% | 98.75% | 98.04% | 97.13% | 88.86% |
| SPN-14-N | 51189318 | 161451839 | 74.34% | 166.84 | 99.48% | 98.92% | 97.89% | 96.18% | 93.67% | 72.62% |
| SPN-14-T | 51189318 | 273613986 | 82.70% | 260.60 | 99.65% | 99.42% | 99.10% | 98.64% | 97.98% | 90.75% |

**Supplementary Table 2.** Non-synonymous somatic mutations identified in SPNs using whole-exome sequencing.

Provided as separate excel file.

**Supplementary Table 3.** Genes in the Wnt pathway assed by Nanostring nCounter.

Provided as separate excel file.

**Supplementary Table 4.** Probes differentially methylation between SPN samples (this study) and ENCODE samples of epithelial origin.

Provided as separate excel file.
